# Demyelination in Multiple Sclerosis: Reprogramming Energy Metabolism and Potential PPARγ Agonist Treatment Approaches

**DOI:** 10.3390/ijms19041212

**Published:** 2018-04-16

**Authors:** Alexandre Vallée, Yves Lecarpentier, Rémy Guillevin, Jean-Noël Vallée

**Affiliations:** 1 Délégation à la Recherche Clinique et à l’Innovation (DRCI), Hôpital Foch, 92150 Suresnes, France; 2 Centre de Recherche Clinique, Grand Hôpital de l’Est Francilien (GHEF), 77100 Meaux, France; yves.c.lecarpentier@gmail.com; 3 Data Analysis and Computations Through Imaging Modeling-Mathématiques (DACTIM), Unité mixte de recherche (UMR), Centre National de la Recherche Scientifique (CNRS) 7348 (Laboratoire de Mathématiques et Application), University of Poitiers, Centre Hospitalier Universitaire (CHU) de Poitiers, 86000 Poitiers, France; remy.guillevin@chu-poitiers.fr; 4 Centre Hospitalier Universitaire (CHU) Amiens Picardie, University of Picardie Jules Verne (UPJV), 80000 Amiens, France; valleejn@gmail.com; 5 LMA (Laboratoire de Mathématiques et Applications), Unité mixte de recherche (UMR), Centre National de la Recherche Scientifique (CNRS) 7348, Université de Poitiers, 86000 Poitiers, France

**Keywords:** WNT/β-catenin pathway, PPARγ, multiple sclerosis, energy metabolism, aerobic glycolysis, demyelination, Warburg effect, circadian rhythms, clock genes

## Abstract

Demyelination in multiple sclerosis (MS) cells is the site of several energy metabolic abnormalities driven by dysregulation between the opposed interplay of peroxisome proliferator-activated receptor γ (PPARγ) and WNT/β-catenin pathways. We focus our review on the opposing interactions observed in demyelinating processes in MS between the canonical WNT/β-catenin pathway and PPARγ and their reprogramming energy metabolism implications. Demyelination in MS is associated with chronic inflammation, which is itself associated with the release of cytokines by CD4^+^ Th17 cells, and downregulation of PPARγ expression leading to the upregulation of the WNT/β-catenin pathway. Upregulation of WNT/β-catenin signaling induces activation of glycolytic enzymes that modify their energy metabolic behavior. Then, in MS cells, a large portion of cytosolic pyruvate is converted into lactate. This phenomenon is called the Warburg effect, despite the availability of oxygen. The Warburg effect is the shift of an energy transfer production from mitochondrial oxidative phosphorylation to aerobic glycolysis. Lactate production is correlated with increased WNT/β-catenin signaling and demyelinating processes by inducing dysfunction of CD4^+^ T cells leading to axonal and neuronal damage. In MS, downregulation of PPARγ decreases insulin sensitivity and increases neuroinflammation. PPARγ agonists inhibit Th17 differentiation in CD4^+^ T cells and then diminish release of cytokines. In MS, abnormalities in the regulation of circadian rhythms stimulate the WNT pathway to initiate the demyelination process. Moreover, PPARγ contributes to the regulation of some key circadian genes. Thus, PPARγ agonists interfere with reprogramming energy metabolism by directly inhibiting the WNT/β-catenin pathway and circadian rhythms and could appear as promising treatments in MS due to these interactions.

## 1. Introduction

Multiple sclerosis (MS) presents chronic inflammation, immune responses, blood–brain barrier (BBB) breakdown, and demyelination in the white matter of the central nervous system (CNS) [1,2]. 

In brain and spinal cord areas, chronic inflammation leads to axonal myelin sheath destruction and the progressive loss of neurological functions with neuronal death. The inflammatory process in MS is initiated by the microglia in association with the release of players CD4^+^ helper (Th) (Th1 and Th17), the markers of the chronic inflammation [3]. Pro-inflammatory mediators, such as cytokines (interleukin (IL-6, IL-17, IL-22), tumor necrosis factor α (TNF-α)), are synthetized by Th17 cells, which are the main immune actors in the pathogenesis of MS [4]. MS can be considered as an autoimmune disease which presents neurological disability and many genetic and environmental determinant etiologies [5].

Glial cells, called oligodendrocytes (OLs), synthetize myelin sheaths in CNS by wrapping axons with multi-lamellar sheets of plasma membrane which are composed of specific lipids and proteins. Loss of myelinating OLs is considered as the origin of MS pathogenesis [6,7,8,9]. In white matter lesions of MS, oligodendrocyte precursor cells (OPC) present a stop state and a non-differentiation into myelinating OLs [6,10,11,12,13,14].

Altered cells in MS are derived from exergonic processes and emit heat that flows to the surrounding environment. Several irreversible processes occur by changing reprogramming energy metabolism [15,16].

Peroxisome proliferator-activated receptor γ (PPARγ) and the WNT/β-catenin pathway act in an opposite manner in many diseases, including MS [17,18]. Numerous autoimmune disorders present this opposed interplay, such as type 1 diabetes [19,20], thyroid autoimmunity [21,22] and rheumatoid arthritis [23,24].

In MS, the dysregulation of both PPARγ [25] and the WNT/β-catenin pathway [26] influence several statistical mechanisms by modifying energy metabolism leading to aerobic glycolysis, called the Warburg effect [27,28].

PPARγ is a member of the nuclear superfamily of ligand-activated transcription factors which regulates glucose metabolism and cellular homeostasis. WNT ligands belong to the family of glycoproteins participating in the control of cell cycle, cell life and embryogenesis. 

The Warburg effect is the shift of an energy transfer production from mitochondrial oxidative phosphorylation to aerobic glycolysis. The Warburg effect was discovered by Otto Warburg in 1930 in cancer processes [28]. This energy shift is partly due to injury of mitochondrial respiration, leading to an increase of adenosine triphosphate (ATP) production by glycolysis. Indeed, although aerobic glycolysis is less efficient in producing ATP molecules than oxidative phosphorylation, its production cycles are much faster than those of oxidation phosphorylation [29], which results in higher ATP molecule production than oxidative phosphorylation [30]. Recent studies have shown that this phenomenon is not specific to cancers but is also observed in non-tumor diseases, such as MS [31]. 

In parallel, dysregulation of circadian rhythms (CRs) has been observed in MS [32]. This dysfunction leads to upregulation of the canonical WNT/β-catenin pathway that contributes to MS pathogenesis. PPARγ can control CRs by regulating some key circadian genes, like Bmal1 (brain and muscle aryl-hydrocarbon receptor nuclear translocator-like 1) [33] and can directly target the WNT pathway [34] and energy balance in CNS [35]. By acting on these systems, PPARγ appears as an interesting therapeutic pathway. In MS, the opposed interplay between PPARγ and the WNT/β-catenin pathway has a major role in the dysregulation of energy metabolism and the disruption of CRs. Several energy balance abnormalities found in MS are induced by several cellular processes involved in both of these. We focus this review on the opposing interactions observed in MS between PPARγ and the canonical WNT/β-catenin pathway and their reprogramming energy metabolism implications.

## 2. PPARγ

Peroxisome proliferator-activated receptor γ (PPARγ) is an orphan nuclear receptor which is a member of the nuclear superfamily of ligand-activated transcription factors [36,37]. PPARγ is composed of a ligand binding domain which is hydrophobic and a type II zinc finger DNA-binding domain [38].

PPARγ ligands form a heterodimer with the retinoic X receptor (RXR). RXR is a 9-*cis* retinoic acid receptor. The heterodimer binds to peroxisome proliferator response element (PPRE) to activate several target genes [39]. PPARγ is highly expressed in adipose tissues [40] and in cardiac and skeletal muscle, pancreatic β-cells, kidney, macrophages [41], and other vascular cells, like endothelial cells [42,43].

PPARγ expression is implicated in numerous homeostasis pathways such as glucose and lipid metabolism. Likewise, PPARγ expression is implicated in migration, apoptosis, cell growth, antioxidant and inflammatory responses [39,44,45]. PPARγ is normally little expressed in CNS [46], but its expression is found in neurons, OLs, astrocytes, microglia/macrophages [47], T and B lymphocytes, dendritic cells [48] and brain endothelial cells [49]. PPARγ can repress inflammation by decreasing nuclear factor-κB (NF-κB) activity [50].

Synthetic ligands of PPARγ are prostaglandins like 15-deoxy-Δ, 14 prostaglandin J2 [51], hydroxyl octadecadienoic acid with derivatives of fatty acid oxidation [52] and lysophosphatidic acid (LPA) [53]. Pioglitazone and rosiglitazone are thiazolidinediones (TZD) which are synthetic PPARγ ligands [52]. 

## 3. Canonical WNT/β-Catenin Pathway (Figure 1)

**Figure 1:**
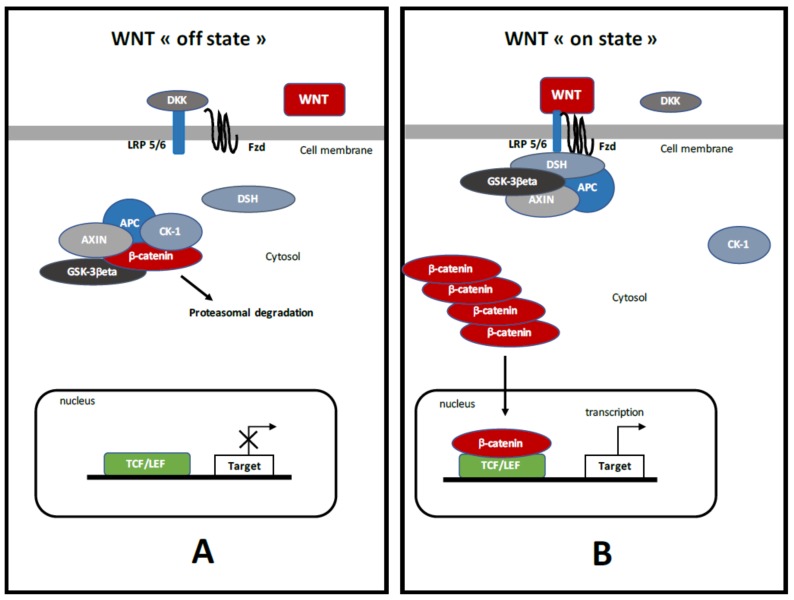
The canonical WNT/β-catenin pathway. (**A**) Under physiological circumstances, the WNT “off state”, the cytosolic β-catenin is bound to its destruction complex, consisting of adenomatous polyposis coli (APC), AXIN and glycogen synthase kinase-3β (GSK-3β). After CK-1 phosphorylates on Ser45 residue, β-catenin is further phosphorylated on Thr41, Ser37, and Ser33 residues by GSK-3β. Then, phosphorylated β-catenin is degraded into the proteasome. Therefore, the cytosolic level of β-catenin is kept low in the absence of WNT ligands. If β-catenin is not present in the nucleus, the T-cell factor/lymphoid enhancer factor (TCF/LEF) complex cannot activate the target genes. Dickkopf (DKK) can inhibit the WNT/β-catenin pathway by binding to WNT ligands or low-density lipoprotein receptor-related protein 5/6 (LRP 5/6). (**B**) When WNT ligands bind to both Frizzled (FZD) and LRP 5/6, the WNT “on state”, Disheveled (DSH) is recruited and phosphorylated by FZD. Phosphorylated DSH in turn recruits AXIN, which dissociates the β-catenin destruction complex. Therefore, β-catenin escapes from phosphorylation and subsequently accumulates in the cytosol. The accumulated cytosolic β-catenin goes into the nucleus, where it binds to TCF/LEF and activates the transcription of target genes.

Canonical WNT/β-catenin pathway is named as the discovery of the cascade gene “W”ingless in drosophila and its homologue in mice “INT”(Integration site) [54] (Figure 1). The WNT/β-catenin pathway is involved in numerous life cycles, such as embryogenesis in migration, proliferation, differentiation, apoptosis and cell polarity [55]. Deregulation of the WNT/β-catenin pathway is observed in several pathologies, such as cancers, fibrosis, neurodegenerative diseases, and atherosclerosis, and its targeting appears as an emerging therapeutic pathway [56].

WNT ligands are glycoproteins, which activate the canonical WNT/β-catenin pathway [57]. Extracellular WNT ligands bind the receptor Frizzled (FZD) and then stimulate the co-receptor Low-Density Lipoprotein (LDL) receptor-related proteins 5 and 6 (LRP 5/6) [58]. 

β-catenin is considered as the main molecule of the canonical WNT pathway. Its major function is transcriptional activity. In physiologic conditions, cytoplasmic β-catenin is in constant turnover between synthetized and destroyed intracellular cycles. 

Cytosolic β-catenin is maintained at a minimal level through the activation of the β-catenin destruction complex, which is formed by a combination of AXIN (a cytoplasmic protein regulating G-protein signaling), glycogen synthase kinase-3β (GSK-3β, a serine-theronine kinase), adenomatous polyposis coli (APC, a tumor suppressor gene), and casein kinase 1 (CK-1, a serine/threonine-selective enzyme) [59]. CK-1 and GSK-3β target β-catenin by phosphorylating the serine and threonine residues located in the amino acid terminus [60,61,62]. CK-1 phosphorylates an N-terminus of β-catenin and GSK-3β phosphorylates a threonine 41 (Th41), Ser33 and Ser37 sites of β-catenin [55,63]. These phosphorylations result in the recruiting of APC in the destruction complex. APC modulates the degradation of the cytosolic β-catenin into the proteasome through its tumor suppressor properties [59,64].

Activation of the WNT/β-catenin pathway is characterized by the initiation of WNT ligands and their interactions with FZD and LRP 5/6 co-receptors [65]. This binding stimulates Disheveled (DSH) to inhibit the destruction complex and to permit cytosolic β-catenin accumulation. Nuclear β-catenin binds T-cell factor/lymphoid enhancer factor (TCF/LEF) to activate several WNT target genes, such as c-Myc and cyclin D1 [66,67]. 

Demyelinating events present an upregulation of the WNT/β-catenin pathway correlated with a release of pro-inflammatory cytokines [68]. Moreover, PPARγ stimulation has a beneficial role in MS [69,70] through the decrease of neuroinflammation [71] and the downregulation of the WNT/β-catenin pathway in MS [17,18]. PPARγ agonists are considered as potential therapeutic perspectives against neuroinflammation and neurodegeneration [72]. In MS, these two pathways operate in an opposed interplay [18] and their dysregulations lead to energy metabolism reprogramming. The objectives of this review are to describe this opposed crosstalk with circadian rhythms regulation, and to better understand the energy remodeling aspect, called the Warburg effect, observed in MS and the potential therapeutic benefits of targeting these two pathways to improve MS-related symptoms.

## 4. Crosstalk between PPARγ and Canonical WNT/β-Catenin Pathway in Diseases

The opposed interplay between the canonical WNT/β-catenin pathway and PPARγ has been observed in numerous pathologies. Cancers, such as gliomas [73,74,75] and colon cancer [76], present an upregulation of the canonical WNT/β-catenin pathway associated with a decrease of PPARγ expression [77]. The process of fibrosis exhibits the same mechanism [78,79,80]. Neurodegenerative diseases are classified in two categories [34], i.e., diseases that present a downregulation of the canonical WNT/β-catenin pathway and an upregulation of PPARγ, such as Alzheimer’s disease [81,82,83], and diseases with an upregulation of the canonical WNT/β-catenin pathway whereas PPARγ is decreased, such as exudative age related macular degeneration [84,85], amyotrophic lateral sclerosis [86], and multiple sclerosis [18].

Numerous studies have suggested that PPARγ may be considered as a negative β-catenin target [87,88]. The β-catenin pathway can decrease PPARγ expression [89,90,91,92,93,94,95,96,97,98]. Indeed, PPARγ and WNT/β-catenin pathway interact via a catenin-binding domain within PPARγ and a TCF/LEF β-catenin domain [99,100,101,102].

The decrease of the WNT/β-catenin pathway stimulates the expression of PPARγ [103], while the increase of PPARγ expression inhibits β-catenin levels in numerous cellular systems [104,105,106]. Troglitazone, a PPARγ agonist, can downregulate c-Myc expression, a WNT target gene [107]. PPARγ agonists, can activate WNT inhibitors, such as Dicckopf-1 (DKK1) [108] and GSK-3β [109] to decrease β-catenin levels. In parallel, the WNT target COUP II can decrease PPARγ [110]. Inflammatory cytokines and cellular pathways, such as WNT/β-catenin pathway, interleukin 1 (IL-1) and TNF-α, can inhibit PPARγ expression [111,112,113].

## 5. PPARγ and the Canonical WNT/β-Catenin Pathway in MS

### 5.1. PPARγ in MS

Several studies have shown that PPARγ agonists can reduce the clinical expression of experimental autoimmune encephalomyelitis (EAE) models of MS (Table 1). In EAE models, the PPARγ agonist 15-deoxy-Δ(12,14)-prostaglandin acts by inhibiting NF-κB activity [114,115,116]. In addition, PPARγ deficiency has been shown to exacerbate the clinical symptoms of EAE models [117]. The downregulation of PPARγ during demyelination in MS is well-described in previous studies [18]. However, the stimulation of PPARγ [118,119] leads to decreased inflammation and permits the remyelination in oligodendrocytes (OLs) models of MS [120]. The overexpression of PPARγ is correlated with neuroprotection in both OLs and neurons [121,122,123,124,125,126,127,128]. Th17 differentiation is decreased by PPARγ agonists in both murine CD4^+^ T cells and in human models [129]. In CNS-infiltrating CD4^+^ T cells, IL-17 expression is diminished by PPARγ overexpression [130]. The anti-inflammatory role of PPARγ is responsible for both the decreased release of inflammatory cytokines [41,131,132], and the expansion of encephalitogenic Th1 [117], Th17 cells [129] and B lymphocytes [133]. Lovastatin induces the expression of PPARγ in the central nervous system (CNS) of EAE models [134]. However, simvastatin impedes the remyelination mechanism in cuprizone-CNS demyelinating models (non-EAE-models) [135,136].

### 5.2. Demyelination and Activation of WNT/β-Catenin Pathway

Several studies have shown that the WNT/β-catenin pathway is overexpressed during the demyelination process (for review, see [18]) (Table 1). The expression of WNT/β-catenin pathway is overexpressed in the spinal cord dorsal horn (SCDH) in EAE models of mice [68]. The increase of the β-catenin inhibitor indomethacin is known to decrease mechanical allodynia in EAE mice [68]. In EAE models, over-activation of the WNT/β-catenin pathway impairs and delays OPC differentiation [137]. The WNT/β-catenin pathway, by stimulating pro-inflammatory cytokines, has a major role in neuropathic pain pathogenesis [138]. β-catenin accumulation and nuclear transcription are associated with alteration of endothelial adherens in experimental models [139,140] and in MS brain tissue [141].

### 5.3. Opposed Interaction between PPARγ and WNT Pathway in MS

In MS models, moringin (4-[α-l-rhamnopyranosyloxy]-benzyl isothiocyanate) can modulate neuroinflammation through both decreased β-catenin signaling and increased PPARγ expression [142]. Moringin can also repress inflammatory factors, such as IL-1, IL-6 and cyclo-oxygenase-2 (COX2) in EAE mice by increasing PPARγ levels [142]. In MS, moringin is known to protect against neurodegenerative disorders [143,144].

## 6. Reprogramming Energy Metabolism in Demyelination

### 6.1. Aerobic Glycolysis 

**Figure 2:**
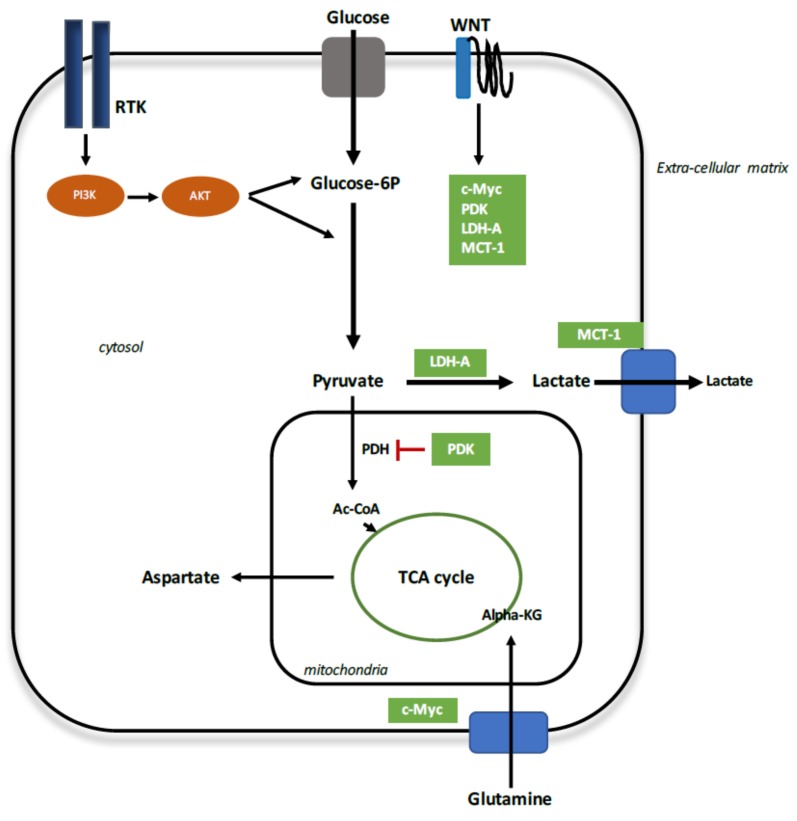
Aerobic glycolysis stimulation by activated canonical WNT/β-catenin pathway. Activation of the receptor tyrosine kinase (RTK) is required to take up enough glucose to cell survival. PI3K/Akt pathway is stimulated to maintain a sufficient ATP production through the metabolism of glucose. Glucose is transformed into pyruvate into the mitochondria for the oxidative phosphorylation process. However, during WNT activation, WNT signal transduction results in activation of c-Myc, lactate dehydrogenase A (LDH-A), pyruvate dehydrogenase kinase (PDK) and monocarboxylate transporter 1 (MCT-1). The WNT target genes cooperate to divert glycolytically derived pyruvate into lactate which is expelled out the cell by MCT-1. Moreover, c-Myc induces glutamine uptake and glutaminolysis to support mitochondrial integrity and aspartate production. Accumulation of cytosolic lactate involves several pathways such as nucleotide synthesis, lipid synthesis and cell division.

Aerobic glycolysis, called the Warburg effect, is the conversion of glucose to lactate in the presence of oxygen sufficient to support glucose catabolism via the tricarboxylic acid (TCA) cycle with oxidative phosphorylation [28] (Figure 2). Numerous studies have shown that the canonical WNT/β-catenin pathway stimulates aerobic glycolysis and glycolytic enzymes such as glucose transporter (Glut), hexokinase (HK), pyruvate kinase M2 (PKM2), lactate dehydrogenase A (LDH-A), monocarboxylate transporter 1 (MCT-1) [27,73,77,85,145,146]. An increased rate of glucose metabolism is correlated with activation of the PI3K/Akt pathway [147]. The WNT/β-catenin pathway directly stimulates PI3K/Akt signaling [148,149]. Activation of the PI3K/Akt pathway leads to HIF-1α stimulation (hypoxia-inducible factor 1-α) [150] to induce overexpression of glycolytic enzymes such as Glut, LDH-A, pyruvate dehydrogenase kinase 1 (PDK1) and PKM2 [150,151]. The allosteric enzyme Phosphofructokinase (PFK) catalyzes the conversion between β-d-fructose-6-phosphate and β-d-fructose-1,6-biphosphate. This reaction, by using ATP, leads to glycolytic oscillations and can be organized in time and space driven by PFK with a positive feedback responsible for periodic behavior [152]. 

### 6.2. Aerobic Glycolysis in MS

An imbalance between energy production and consumption has been observed in MS [153,154,155]. Decrease of oxidative phosphorylation and mRNA deletions observed in MS neuronal cell bodies indicate a mitochondrial dysregulation [156,157]. Indeed, alteration of energy metabolism is observed in urine [158] and in serum of MS patients [159]. Activation of aerobic glycolysis and decrease of oxidative phosphorylation aggravate MS pathogenesis by inducing dysfunction of CD4^+^ T cell [160]. CD4^+^ T cell dysregulation has a major role in MS pathogenesis by aggravating axonal and neuronal damage [1,161]. 

Inhibition of aerobic glycolysis in MS by copaxone restores mitochondrial activity and then diminish CD4^+^ T cell dysregulation [162]. Glycolytic metabolism reduces ROS (reactive oxygen species) production, oxidative damage and promotes the production of lipids and fatty acid required by OLs for myelin production [163,164,165].

Neuronal cell death and astrocytic inflammation processes are associated with the increase of glycolytic activity [166,167]. Shunt of TCA cycle by decrease of pyruvate dehydrogenase (PDH) activity is associated with neurodegeneration [168,169]. Lactate metabolism is upregulated upon the increase of aerobic glycolysis in MS [170,171]. The increase in lactate levels is correlated with the progression of MS [172,173]. Reduction of oxidative phosphorylation, shunt of the TCA cycle and activation of aerobic glycolysis inducing lactate production are observed in MS lesions [31,159]. Recently, magnetic resonance spectroscopy and positron emission tomography (PET) have shown that lactate levels are increased in MS lesions [170,174] and that lactate concentration in the cerebral spinal fluid is associated with the number of inflammatory plaques and mitochondrial dysregulation in MS [175,176,177]. Modulation of aerobic glycolysis appears as a potential treatment for myelin maintenance in MS lesions [26].

## 7. Circadian Rhythms in MS

### 7.1. Circadian Rhythms, Definition

**Figure 3:**
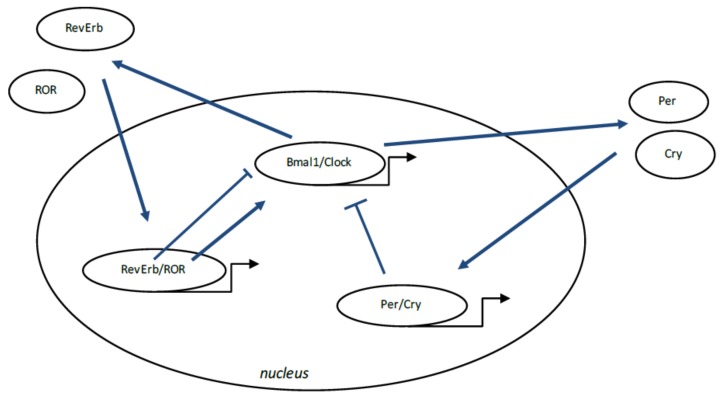
Circadian clock genes process. The clock is considered as a stimulatory loop, with the Bmal1/Clock heterodimer activating the transcription of Period (Per) and Cryptochrome (Cry) genes, and then a negative feedback loop with the Per/Cry heterodimer which translocates to the nucleus and then represses the transcription of the Clock and Bmal1 genes. An additional loop implicates the RORs and Rev Erbs factors with a positive feedback by retinoid-related orphan receptors (ROR) and a negative feedback by Rev Erbs. Arrows: activation; T bar: inhibition.

Several biologic mechanisms in the body are controlled by the circadian “clock” (circadian locomotors output cycles kaput). The circadian clock is located in the hypothalamic suprachiasmatic nucleus (SCN). CRs are endogenous and entrainable free-running periods that last approximately 24 h. Numerous transcription determinants are responsible for the regulation of CRs. They are called circadian locomotor output cycles kaput (Clock), brain and muscle aryl-hydrocarbon receptor nuclear translocator-like 1 (Bmal1), Period 1 (Per1), Period 2 (Per2), Period 3 (Per3), and Cryptochrome (Cry 1 and Cry 2) [178,179] (Figure 3). These transcription factors are controlled by positive and negative feedbacks mediated by CRs [180,181]. Clock and Bmal1 heterodimerize and then initiate transcription of Per1, Per2, Cry1 and Cry2 [182]. The Per/Cry heterodimer can inhibit its activation through negative feedback. It translocates back to the nucleus to directly inhibit the Clock/Bmal1 complex and then inhibits its own transcription [182]. 

The Clock/Bmal1 heterodimer activates the transcription of retinoic acid-related orphan nuclear receptors, Rev-Erbs and retinoid-related orphan receptors (RORs). By a positive self-regulation RORs can activate Bmal1 transcription, whereas Rev-Erbs can repress their transcription through negative feedback [182]. 

### 7.2. Circadian Rhythm Disruption in MS

Several studies have shown that circadian rhythms have a main role in MS [183]. Late-night shift work in MS patients is associated with disruption of circadian rhythms and sleep [184,185]. Indeed, sleep dysregulation worsens EAE symptoms [186] by increasing the infiltration of inflammatory cells in the CNS, such as CD4^+^ T cells [186]. EAE severity is associated with both sleep disruption and degree of sleep fragmentation [187]. Few studies have examined CRs dysregulation in MS. However, MS is associated with many symptoms such as hypertension, heart disease, anxiety, depression and sleep disturbances [188,189]. Sleep disorders observed in MS patients [190] are characterized by prolonged sleep latency and frequent nocturnal awakening [191]. In MS, fatigue symptom is associated with CRs abnormalities [192,193], such as sleep phase syndrome and irregular sleep wake pattern. In MS, dorsolateral hypothalamic neurons secrete less neuropeptide hypocretin-1/orexin-A [194]. The Orexin-A system is responsible for the modulation of sleep-wake cycle [195]. Decreased orexin-A levels lead to the promotion of consolidated night sleep [196]. 

Hypersomnia observed in MS patients is associated with low cerebrospinal fluid (CSF) orexin-A and hypothalamic lesions [197]. Inflammation may suppress the orexin-A system [198] through the overexpression of cytokines, TNF-α and interferon γ (IFN-γ) leading to fatigue syndrome in MS patients [199]. 

The orexin-A system is influenced by seasonal fluctuations and day length [200]. Demyelination process may put MS patients at risk for CR disorders [201]. Seasonal fluctuations observed in MS may be due to variations of melatonin levels which increase in winter and decrease in summer [202]. Moreover, this seasonal variation could also act through the birth month in susceptibility to developing MS [203,204]. Cytokine and chemokine expression in lymphoid tissues present some seasonal variation in EAE mice [204,205]. 

By inducing Rev-Erbs, CRs can regulate the balance of Th17/Th1/Treg in EAE mice [206]. The number of Th17 cells decreases during the acute phase of MS and is associated with melatonin levels [206]. 

### 7.3. Interaction between WNT/β-Catenin Pathway and Circadian Rhythms

**Figure 4:**
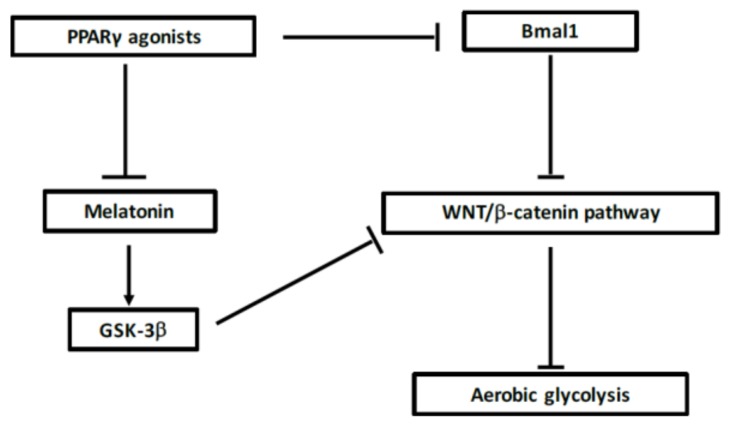
Schematic interaction between WNT, peroxisome proliferator-activated receptor γ (PPARγ) and circadian rhythms. PPARγ agonists can decrease Bmal1 expression. The knockout of Bmal1 leads to decrease WNT/β-catenin pathway activity and then in absence of initiation of aerobic glycolysis. In parallel, PPARγ agonists can decrease melatonin levels leading to activate GSK-3β activity, the main inhibitor of WNT pathway.

The WNT/β-catenin pathway is downstream of the RORs regulation factors and contains diverse putative Bmal1 clock-binding sites within its promoter [207]. By these interactions, circadian genes can regulate cell cycle progression through the WNT pathway [208] (Figure 4). Expression of WNT pathway can be downregulated by Bmal1 knockdown [209]. Expression levels of WNT-related genes in wild-type mice are higher than levels of WNT-related genes with Bmal1 knockdown mice [210,211]. Bmal1 appears to be upregulated in MS [212]. Cell proliferation and cell cycle progression are regulated by Bmal1 via stimulation of the canonical WNT/β-catenin pathway [213]. Bmal1 activation increases β-catenin transcription and decreases both β-catenin degradation and GSK-3β activation [214]. Per2 degradation induced by β-catenin involves the dysregulation of circadian genes in intestinal mucosa of ApcMin/+ mice [215]. 

In normal circumstances, the core circadian genes work in accurate feedback loops and keep the molecular clockworks in the SCN. They permit regulation of peripheral clocks [180,181]. 

Per1 and Per2 control CRs cells and modulate cell-related genes expression, such as c-Myc, to sustain the normal cell cycle [216,217]. mRNAs and proteins levels of circadian genes oscillate throughout the 24-hour period [180]. 

### 7.4. Action of PPARγ on Circadian Rhythms

PPARγ directly acts with the core clock genes and presents diurnal fluctuations in liver and blood vessels [33,218]. In mice, impaired diurnal rhythms are induced by a knockdown of PPARγ [219]. PPARγ agonists can regulate Bmal1 and the constitution of the heterodimer Clock/Bmal1 [33,220] and can then target Rev-Erb [221] (Figure 4). Decrease of the clock-controlled gene Nocturin inhibits PPARγ oscillations in the liver of mice fed on high-fat diet. In normal circumstances, nocturin binds to PPARγ to enhance its transcriptional activity [222]. The inhibtion of PPARγ expression prevents circadian function of 15-Deoxy-D 12,14-prostaglandin J2 (15-PGJ2) [219]. The partner of PPARγ, RXR, interacts with Clock protein in a ligand-dependent manner and then decreases the formation and transcriptional activity of the Clock/Bmal1 heterodimer [223]. PPARγ acts on the mammalian clock and energy metabolism [223]. Circadian metabolism is directly regulated by PPARγ [219]. Retinoic acid receptor-related orphan receptor γ t (RORγt) is considered as a key transcriptional factor for Th17 differentiation [224,225]. PPARγ can influence the function of Th cell clones [226]. PPARγ agonists inhibit Th17 differentiation through the inhibition of RORγt induction [129,227,228]. CD4^+^ T cells fail to express RORγt under the action of PPARγ agonists [129].

### 7.5. Interest of Cortisol in MS

Cortisol production by the HPA axis (hypothalamic-pituitary-adrenal axis) during the acute phase of MS leads to suppression of T cell secretion of inflammatory factors, such as cytokines [229]. The peak of inflammatory factor production occurs in association with low levels of cortisol. TNF-α and IL-6 production during active phases is consistent with reduction of night-time cortisol production [230]. Cortisol production is regulated by circadian rhythms that present an elevated morning level, but normalizes by the evening in MS patients [231]. However, the role of cortisol in MS remains unclear [183]. HPA axis seems to be activated in relapsing-remitting MS patients but this phenomenon has not been shown in all studies [232,233]. Nevertheless, a normal cortisol level is associated with a more severe disease course [234]. Rat strains with low HPA axis activity present worse EAE in comparison to rat strains with low HPA axis activity [235]. These results suggest that elevated levels of cortisol suppress inflammation in MS even if other studies have shown that over-active HPA axis in association with high serum cortisol contributed to worse forms of MS [236]. Cortisol seems not to act alone, and corticosteroids present in the CNS and blood may have an impact on the immune response. Corticosteroid concentration in cerebrospinal fluid present high levels in MS patients with stable disease and low levels with worse forms despite similar serum cortisol level [237]. High serum cortisol level can inhibit inflammation process through an over-active HPA axis leading to protection in MS whereas disruption of HPA axis is associated with worse forms of the disease. Cortisol is known to have immunosuppressive effects by affecting cytokine secretion and T cell activation [238]. Glucocorticoids are not limited to the inhibition of T cell response but also affect the decrease of macrophages, B cells and dendritic cells [239]. Glucocorticoids in MS present many beneficial effects but the resistance observed in humans may complicate its use [240]. Nevertheless, cortisol can affect CRs by glucocorticoid receptors [by activating the transcription of Per1 and Per2 [241,242]. In MS, the circadian oscillations of cortisol levels show that cortisol may have a key role in the regulation of peripheral clocks. In MS, no study has shown a link between PPARγ expression and cortisol level. Few studies have observed that high PPARγ agonists can increase cortisol levels in cancers [243]. 

### 7.6. Interest of Melatonin in MS

Melatonin (also named 5-methoxy-*N*-acetyltryptamine) is a secreted by the pineal gland [244]. Melatonin is released during darkness and thereby regulates the circadian regulation of sleep [245,246]. An inverse correlation is observed between melatonin levels and MS progression [247,248]. Melatonin has anti-inflammatory, anti-oxidant and neuroprotective effects [245,249,250,251,252,253]. Administration of melatonin reduces EAE severity through the suppression of Th17 cell number [202,254]. Moreover, CRs could be related to inflammation by affecting immunization [255]. TNF-α and IL-1β overexpression can inhibit the melatonin synthesis pathway [256,257,258]. TNF-α directly inhibits melatonin expression [259]. Melatonin ameliorates EAE development by suppressing Th17 cells generation [202,254,260,261,262]. Melatonin also ameliorates symptoms in EAE mouse models [202,254,260,261,262] through the inhibition of Rev-Erb and ROR expressions, and by limiting Th17 cell differentiation and function [206,260]. Melatonin decreases phosphorylation of GSK-3β [263,264]. PPARγ agonists can upregulate melatonin levels to restore mitochondrial membrane potential, stimulate the biogenesis of mitochondria [265] and enhance mitochondrial function [266]. 

## 8. Conclusions

**Figure 5:**
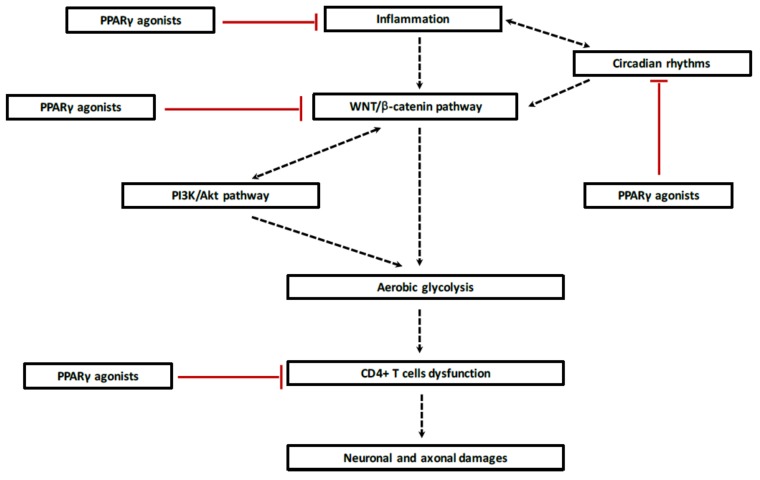
Potential PPARγ agonists treatment approach in demyelination. During acute phase, inflammation processes, activated by disruption of circadian rhythms, lead to release of several cytokines and pro-inflammatory factors which stimulate the canonical WNT/β-catenin pathway. Activation of the WNT ligands involves WNT target genes that are responsible for the initiation of the shunt of the tricarboxylic acid (TCA) resulting in aerobic glycolysis instead of oxidative phosphorylation. Lactate production, the main factor of energy metabolism alteration, and its release out the cells enhance CD4^+^ T cells dysfunction which aggravates MS pathogenesis, neuronal and axonal damages. Using PPARγ agonists could be interesting because of their four interactions in the demyelination cascade. First, PPARγ agonists directly inhibits neuroinflammation by inhibiting cytokines and inflammatory factors release. Secondly, PPARγ agonists can regulate circadian clocks, such as Bmal1, to decrease inflammatory factors release and to target WNT ligands. Third, their opposed interaction with the canonical WNT/β-catenin pathway can prevent the initiation of aerobic glycolysis process and then the energy metabolism reprogramming enable MS. At last, PPARγ agonists have neuroprotective effects by targeting CD4^+^ T cells to prevent neuronal and axonal damages. Arrow: activation; T bar: inhibition.

Demyelination during MS lesions is associated with reprogramming energy metabolism through the dysregulation of the opposed interplay of PPARγ and the WNT/β-catenin pathway (Table 1). The canonical WNT/β-catenin pathway is upregulated by chronic neuroinflammation, whereas PPARγ is downregulated during demyelinating processes. These two systems act in an opposed and reverse manner. Demyelinating processes are associated with the increase of the WNT/β-catenin pathway and dysregulation of the circadian clock genes. In MS, over-activation of Bmal1 leads to stimulation of the canonical WNT/β-catenin pathway. Then, activation of the WNT/β-catenin pathway results in stimulation of glycolytic enzymes leading to activation of aerobic glycolysis. Lactate production induces dysfunction of CD4^+^ T cells leading to axonal and neuronal damage during the MS demyelinating processes. PPARγ agonists can inhibit Th17 differentiation in CD4^+^ T cells and can diminish cytokine release. In parallel, PPARγ agonists can interfere with reprogramming energy metabolism by directly inhibiting the WNT/β-catenin pathway and interacting with clock genes and thus, could be a promising therapeutic pathway in MS due to their interactions (Figure 5). These findings support the possibility of targeting these pathways with the goal of improving the symptoms of MS. Clinical trials and studies are needed to confirm this hypothesis in MS pathogenesis.

## Figures and Tables

**Table 1:** WNT pathway, peroxisome proliferator-activated receptor γ (PPARγ) and aerobic glycolysis in multiple sclerosis (MS) models.

Pathway	Expression	Actions	Model	References
**PPARγ**	Agonists	Inhibition of NF-κB	EAE models	[114,115,116]
		Decrease inflammation, permits remyelination	OLs models	[118,119]
		Neuroprotection	EAE models	[121,122,123,124,125,126,127,128]
		Th17 differentiation	Murine CD4^+^ T cells	[129]
		Decrease IL-17 expression	EAE models	[130]
		Decrease IL-1, IL-6 and COX2	EAE models	[142]
		Decrease β-catenin	EAE models	[142]
**WNT**	Overexpression	Chronic pain	EAE models	[68]
		Impairs OPC differentiation	EAE models	[137]
		Alteration of endothelial adherens	EAE models	[139,140]
		Alteration of endothelial adherens	MS brain tissue	[141]
**Aerobic Glycolysis**	Activation	Neuronal cell death and astrocytic inflammation	EAE models	[166,167]
		MS progression	Human models	[172,173]
		Increased lactate production	Human models	[31,159]
		Mitochondrial dysregulation	Human models	[175,176,177]

NF-κB: nuclear factor-κB; EAE: experimental autoimmune encephalomyelitis; OLs: oligodendrocytes; OPC: oligodendrocyte precursor cells; MS: multiple sclerosis.

## Abbreviations

Acetyl-coA	Acetyl-coenzyme A
APC	Adenomatous polyposis coli
Bmal1	Brain and muscle aryl-hydrocarbon receptor nuclear translocator-like 1
Clock	Circadian locomotor output cycles kaput
Cry	Cryptochrome
CRs	Circadian rhythms
DSH	Disheveled
FZD	Frizzled
Glut	Glucose transporter
GSK-3β	Glycogen synthase kinase-3β
LDH	Lactate dehydrogenase
LRP 5/6	Low-density lipoprotein receptor-related protein 5/6
MCT-1	Monocarboxylate lactate transporter-1
Per	Period
PPARγ	Peroxisome proliferator-activated receptor γ
PI3K-Akt	Phosphatidylinositol 3-kinase-protein kinase B
PDH	Pyruvate dehydrogenase complex
PDK	Pyruvate dehydrogenase kinase
RORs	Retinoid-related orphan receptors
TCF/LEF	T-cell factor/lymphoid enhancer factor
TCA	Tricarboxylic acid
